# A novel, non-radioactive eukaryotic *in vitro* transcription assay for sensitive quantification of RNA polymerase II activity

**DOI:** 10.1186/1471-2199-15-7

**Published:** 2014-04-03

**Authors:** Cristina Voss, Brita Schmitt, Susanne Werner-Simon, Christian Lutz, Werner Simon, Jan Anderl

**Affiliations:** 1 Department of Biochemistry and Cell Biology, Heidelberg-Pharma GmbH, Schriesheimer Str. 101, Ladenburg D-68526, Germany; 2 Department of Chemistry, Heidelberg-Pharma GmbH, Schriesheimer Str. 101, Ladenburg D-68526, Germany; 3 Department of Analytical Chemistry, Heidelberg-Pharma GmbH, Schriesheimer Str. 101, Ladenburg D-68526, Germany

## Abstract

**Background:**

Many studies of the eukaryotic transcription mechanism and its regulation rely on *in vitro* assays. Conventional RNA polymerase II transcription assays are based on radioactive labelling of the newly synthesized RNA. Due to the inefficient *in vitro* transcription, the detection of the RNA involving purification and gel electrophoresis is laborious and not always quantitative.

**Results:**

Herein, we describe a new, non-radioactive, robust and reproducible eukaryotic *in vitro* transcription assay that has been established in our laboratory. Upon transcription, the newly synthesized RNA is directly detected and quantified using the QuantiGene assay. Alternatively, the RNA can be purified and a primer extension followed by PCR detection or qPCR quantification can be performed. When applied to assess the activity of RNA polymerase II inhibitors, this new method allowed an accurate estimation of their relative potency.

**Conclusions:**

Our novel assay provides a non-radioactive alternative to a standard *in vitro* transcription assay that allows for sensitive detection and precise quantification of the newly transcribed, unlabelled RNA and is particularly useful for quantification of strong transcriptional inhibitors like α-amanitin. Moreover, the method can be easily adapted to quantify the reaction yield and the transcription efficiency of other eukaryotic *in vitro* systems, thus providing a complementary tool for the field of transcriptional research.

## Background

A tight regulation of gene expression is crucial for the development of an organism and the maintenance of cellular homeostasis, while aberrant gene expression leads to disease-related altered phenotypes. The control of transcription plays herein a key role, and research is still ongoing to decipher its mechanisms. Many of these studies rely on *in vitro* RNA polymerase II transcription assays [1-3].

**Figure 1:**
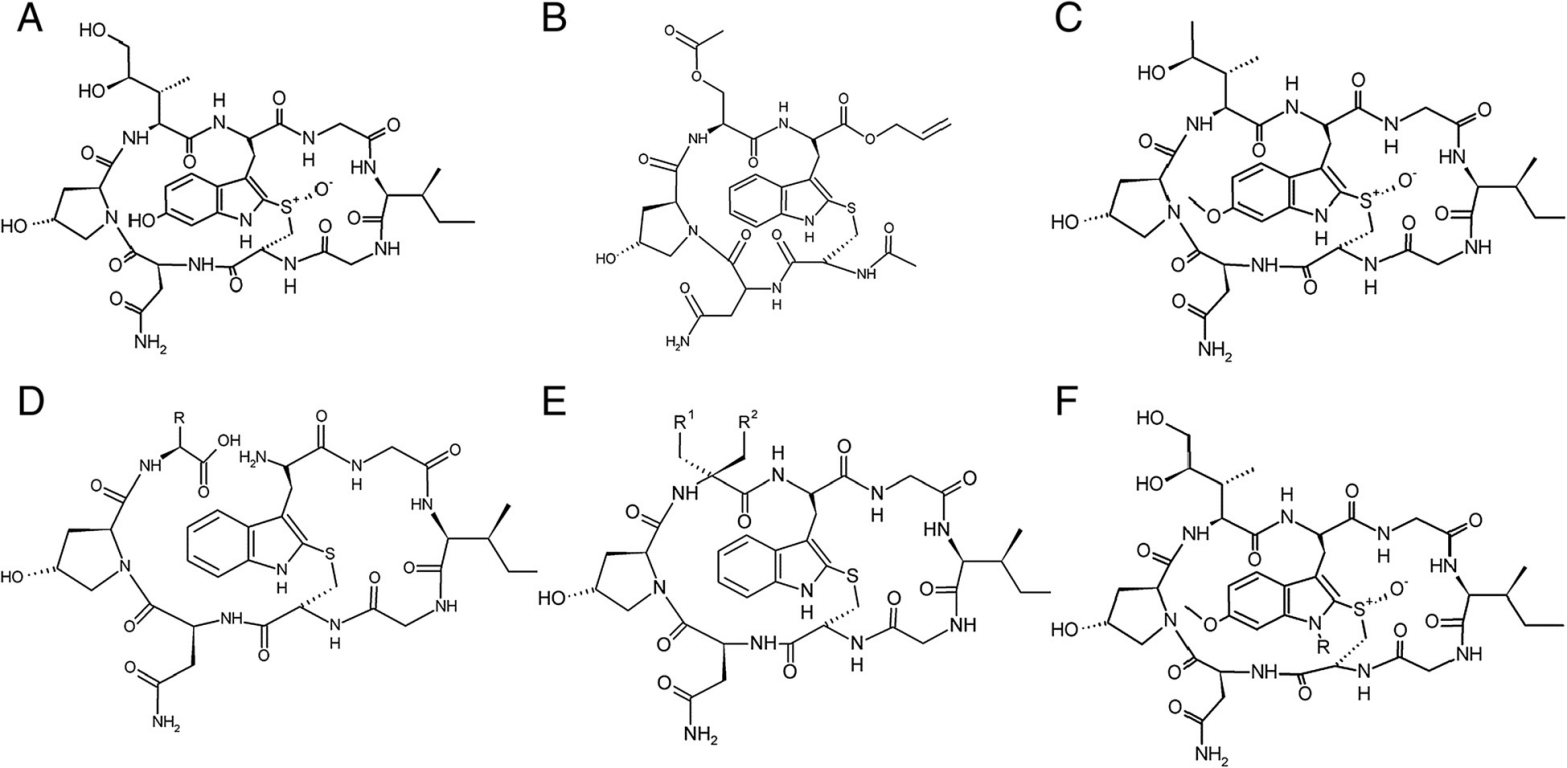
**Chemical structure of α-amanitin and amanitin-analogs tested in this paper. A**. α-amanitin. **B**. O-methyl-γ-amanitin. **C**. HDP30.0378 [R = (CH_2_)_6_-NH_2_], HDP30.0516 [R = (CH_2_)_8_-NH_2_] and HDP30.0592 [R = (CH_2_)_4_-NH_2_]. **D**. HDP30.0346. **E**. HDP30.0445 [R = CH(CH_3_)–CH_2_-CH_3_] and HDP30.0528 [R = CH_2_-OH]. **F**. HDP30.0470 [R^1^ = CH(CH_3_)–CH_2_-CH_3_, R^2^ = H] , HDP30.0797 [R^1^ = phenyl, R^2^ = H], HDP30.0841 [R^1^ = 4-flour-phenyl, R^2^ = H], HDP30.0890 [R^1^ = 4-hydroxy-phenyl, R^2^ = H] and HDP30.0931 [R^1^ = CH_2_-OH, R^2^ = CH_2_-OH].

Effective transcription is especially critical for transformed cells [4]. Since inhibition of transcription leads to apoptosis regardless of the p53 status of the cells, interfering with transcription is a promising therapeutic strategy for developing new anticancer agents [5,6]. Several common chemotherapeutic agents indirectly inhibit translation by damaging the DNA (cisplatin), by topomerase inhibition (camptotecin, doxorubicin) or by inhibiting RNA polymerase II activation via cyclin-dependent kinase inhibition (flavopyridol). Compounds that directly inhibit the RNA polymerase II as the mushroom-derived amanitins (Figure 1A) are well-known toxins [7,8]. However, in combination with antibodies targeting specific cancer antigens they turn into potent and specific antineoplastic agents [9,10]. For evaluation of the efficacy of new transcription inhibitors derived from medicinal chemistry approaches, the quantification of inhibitory activity is necessary. The activity of different compounds can be best compared using a standardized *in vitro* polymerase II transcription run-off assay [11,12].

For a typical polymerase II run-off reaction, a well-defined nuclear extract providing the RNA polymerase II and a basal set of transcription factors are used together with a linear DNA template containing the desired promoter. The newly synthesized RNA is radioactively labeled by adding a ^32^P-CTP to the reaction mixture. After the transcription reaction, the RNA has to be detected and/or quantified. Most commonly, the RNA is purified by phenol-chloroform extraction and ethanol precipitation. An RNA gel electrophoresis is performed and the labeled RNA detected by autoradiography [1,2]. Alternatively, when unlabeled, the purified RNA can be detected by primer extension using a fluorescently labeled primer [13]. After reverse transcription, the resulting cDNA is purified and a gel electrophoresis performed.

However, these methods are difficult to use for an unexperienced laboratory. Since the synthesized amounts are minute, reproducible and quantitative RNA recovery during purification is challenging and strongly depends on the experience of the laboratory staff. Quantification of the newly synthesized RNA requires a phosphor-imaging device. Moreover, because of the laborious and time-consuming procedure, studies involving high numbers of probes are difficult.

To overcome these limitations, our laboratory has developed a non-radioactive *in vitro* transcription assay that relies on a commercially available eukaryotic transcription kit and quantitative PCR RNA detection. Moreover, the method was further optimized by using a novel hybridization method for RNA detection and quantification. With this optimized detection the purification step can be avoided so that the method can be used for the concomitant analysis of a considerable number of samples including replicates. We applied both new methods to quantify the activity of the strong RNA polymerase II direct inhibitor α-amanitin vs. natural, synthetic and semisynthetic amanitin-derivatives (Figure 1). Moreover, we employed the methods for quantification of transcriptional activity from a promoter lacking the TATA box, as well as of the inhibitory activity of flavopiridol, which affects transcription by binding to the P-TEFb kinase.

## Results

### Primer extension followed by PCR detection

**Figure 2:**
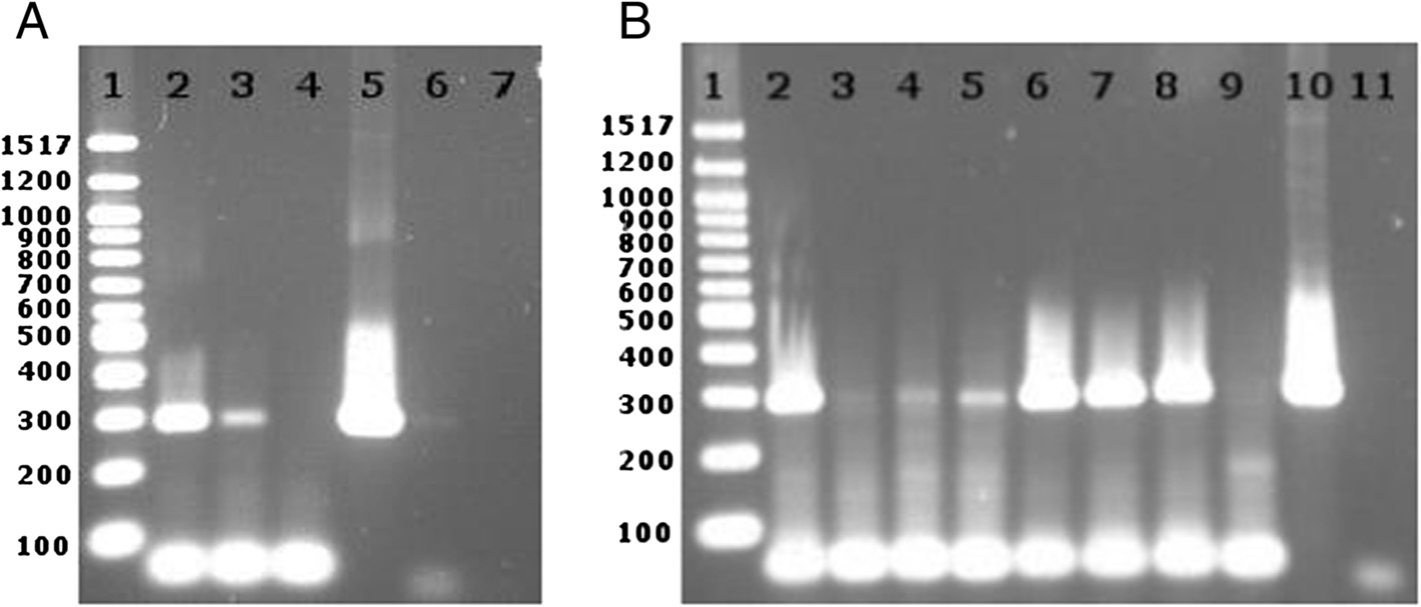
**Detection of newly synthesized RNA by primer extension and PCR.***In vitro* transcription was performed on the pEGFP-N1 plasmid **(A)** or linearized CMV-EGFP template **(B)**. The RNA was purified, reverse transcribed and detected by PCR. **A**. Gel analysis of the PCR products demonstrating α-amanitin-sensitive RNA synthesis. 1- DNA ladder; 2- no α-amanitin; 3- 100 μM α-amanitin; 4- negative transcription control (without NTPs); 5- positive PCR control; 6- DNase digested DNA control; 7- water PCR control. **B**. The visualized PCR product amount inversely correlated with the α-amanitin concentration. 1- DNA ladder; 2- no α-amanitin; 3-5- 400, 100 and 20 μM α-amanitin, respectively; 6-8- 400, 100 and 20 μM synthetic amanitin analog 30.0346, respectively; 9- negative transcription control (without NTPs); 10- positive PCR control; 11-water PCR control.

For the first transcription followed by PCR detection experiments, the well-characterized plasmid pEGFP-N1 (Clontech, acc. no. U55762) was directly used as a template. The reactions were set up using the HeLa Scribe kit without adding radioactive nucleotides. To assess transcription inhibition, α-amanitin or other compounds were added in various concentrations to the reaction mix. A transcription reaction mixture that did not include NTPs served as a negative control, since it contained the same amount of template DNA and would thus monitor DNA interference. After transcription, the newly synthesized RNA was purified and DNase digested. For RNA detection, a primer pair was designed and used in a reverse transcription plus PCR approach to amplify a 295 bp long DNA stretch within the first 400 bp of the EGFP transcript (Table 1). Figure 2A shows the agarose gel of the amplified products. A DNA fragment of expected length was synthesized using the RNA from the positive transcription reaction, while RNA from the reaction without NTPs showed no product, proving that the PCR product was transcription-dependent. Moreover, addition of α-amanitin to the transcription mix resulted in a clearly diminished amount of PCR product.

**Table 1:** Primer and probe sequences for amplification and detection

**Template**	**Primer/oligo**	**Sequence 5′-3′**	**Modification**	**Amplicon/probe**
Plasmid pEGFP-N1	CMV-EGFP-frw	GGGGCGGAGCCTATGGAAAA	-	PCR product: (5′ biotinyl-)CMV-EGFP, 1136 bp
	Btn-CMV-EGFP-frw	GGGGCGGAGCCTATGGAAAA	5′ biotin	
	CMV-EGFP-rev	TGTCGCCCTCGAACTTCACCTC	-	
CMV-EGFP	EGFPfrw1	TGAGCAAGGGCGAGGAGCTGTT	-	PCR/qPCR product, 295 bp
	EGFPrev1	AAGATGGTGCGCTCCTGGACGT	-	
	EGFPfrw2	GTGACCACCCTGACCTAC	-	qPCR product, 83 bp
	EGFPrev2	ATGGCGGACTTGAAGAAG	-	
	EGFP-LNA1	CAGtGCtTCaGCcGCTA*	5′ FAM, 3′ BHQ1, LNA-oligo*	qPCR dual labeled probe
HeLaScribe ‘Positive Control DNA’**	HELA NUCLEAR FW	CTCATGTTTGACAGCTTATCGATCCGGGC	-	PCR product: (5′ biotinyl-)HS-DNA, 1182 bp
	Btn-HELA NUCLEAR FW	CTCATGTTTGACAGCTTATCGATCCGGGC	5′ biotin	
	HELA NUCLEAR RV	ACAGGACGGGTGTGGTCGCCATGAT	-	
HS-DNA	HNqPCRfrw1	GCCGGGCCTCTTGCGGGATAT	-	qPCR product, 132 bp
	HNqPCRrev1	CGGCCAAAGCGGTCGGACAGT	-	
	HNqPCRfrw6	GTCCATTCCGACAGCATCGCCA	-	qPCR product, 144 bp
	HNqPCRrev6	GGCTCCAAGTAGCGAAGCGAGC	-	
	HN_SONDE1	TGGCGTGCTGCTAGCGCTAT	5′ FAM, 3′ BHQ	qPCR dual labeled probe

*Locked Nucleic Acid (LNA) - bases are depicted as lower case.

**‘Positive Control DNA’ is provided with the Promega ‘HeLaScribe Nuclear Extract *in vitro* Transcription System’. Its DNA sequence is provided within the Additional file 1 “HeLaScribe Positive Control DNA Sequence”.

The residual transcriptional activity that was observed in the presence of 100 μM α-amanitin was attributed to RNA polymerases I or III recognizing alternative bacterial promoters on the plasmid. To minimize this interference, CMV-EGFP, a 1136 bp linear DNA template containing the CMV promoter and about 470 bp of the EGFP transcript, was generated by PCR amplification. Transcription reactions with this linear template followed by the primer extension & PCR for detection method showed gradually increasing inhibitory effects of increasing α-amanitin concentrations (Figure 2B). In contrast, a synthetic α-amanitin derivative (HDP30.0346, Figure 1B) containing only the left ring of the α-amanitin bicyclic octapeptidic structure showed in this experiment no inhibitory activity.

### Primer extension with qPCR quantification

**Figure 3:**
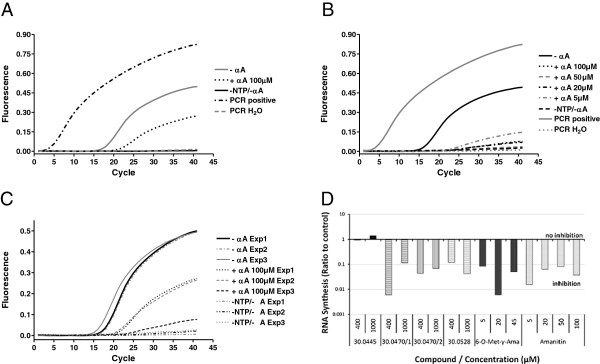
**Detection of newly synthesized RNA by primer extension and qPCR.***In vitro* transcription was performed on the pEGFP-N1 plasmid **(A, C)** or linearized CMV-EGFP template **(B, C, D)**. The RNA was purified, reverse transcribed and detected by qPCR. **A**. qPCR amplification curves of the primer-extension products from A as detected by a sequence-specific LNA probe. The C(T) value of the α-amanitin-inhibited probe (+αA 100 μM) of 22.3 was significantly lower than that of the probe generated without amanitin (−αA, c(T) = 17.6) indicating significantly lower RNA amounts. **B**. qPCR amplification of RNA/cDNA after *in vitro* transcription from the linear template CMV-EGFP in the presence of 5-100 μM α-amanitin showed no clear α-amanitin-concentration dependency. -αA – positive transcription (without α-amanitin, C(T) = 16.1); +αA – α-amanitin-inhibited transcription, C(T) values 27.9, 37.0, 28.1 and 24.5 for 100, 50, 20 and 2 μM, respectively; −NTP/-αA – negative control transcription; DNase - DNase digested DNA control. **C**. Amplification curves of RNA products from three different experiments (Exp1-Exp3). Positive transcription reactions performed at different occasions resulted in very similar C(T) values of 17.6, 18.0 and 16.1 for Exp1, 2 and 3, respectively. No amplification occurred for the negative controls. Note that the inhibitory effect of α-amanitin was dramatically increased when the linearized template CMV-EGFP was used for transcription (Exp3, C(T) = 27.9), when compared to Exp1 and Exp2 (C(T) values 22.3 and 21.7, respectively), were the supercoiled plasmid pEGP-N1 was used as a template. αA – transcription without α-amanitin; +αA - transcription in the presence of α-amanitin; −NTP/-αA – negative control transcription. **D**. Semi-quantitative estimation of the relative inhibitory activity of α-amanitin and several semisynthetic and synthetic analogs. 6–O-Met-γ-Ama = 6′-O-methyl-γ-amanitin; Amanitin = α-amanitin. Two different charges of the analog HDP30.0470 were tested.

To quantitate the observed gradual inhibition by α-amanitin, the established PCR was replaced by a standard qPCR with SYBR green quantification. Despite the promising PCR results, in a first qPCR experiment no differences were observed, although the same RNA samples and primers were used. The product melting curve analysis showed that unspecific products interfered with the quantification. SYBR dye-based quantification was thus replaced by quantification via the dual-labeled sequence-specific probe *EGFP-LNA1* (Table 1). *EGFP-LNA1*-based quantification revealed a clear difference between the positive, negative and inhibited reactions (Figure 3A). However, the efficiency of the qPCR reactions dropped significantly upon using the probe, and no α-amanitin-concentration dependency could be discerned even when using the linear CMV-EGFP fragment as a template (Figure 3B).

On the other hand, when comparing RNA samples derived from different *in vitro* transcription experiments, a very good reproducibility of RNA detection and quantification was observed (Figure 3C). RNA samples from different translation reactions performed at different occasions showed very similar fluorescence levels and C(T) values. Interestingly, the inhibitory effect of 100 μM α-amanitin was clearly stronger in reactions performed with the linear DNA template (Experiments 1 and 2), when compared to the transcription reactions on the circular plasmid template (Experiment 3).

This qPCR setting was subsequently used to compare the inhibitory activity of α-amanitin and several analogs in a single experiment. The methylated amanitin variant 6′–O-methyl-γ-amanitin (Figure 1C) was used in a concentration range similar to α-amanitin (5-100 μM), while the concentrations of synthetic ones were chosen 10-fold higher (400-1000 μM). For quantification, a standard curve of the linear template CMV-EGFP was used. The results of this experiment demonstrated that this method allowed for a semi-quantitative comparison of α-amanitin and analogs (Figure 3D). 6′–O-methyl-γ-amanitin, an about two-fold less potent RNA polymerase II inhibitor than α-amanitin [14], showed an inhibitory activity similar to that of α-amanitin in our setting. One of the synthetic compounds, HDP30.0445 (Figure 1D), an α-amanitin-analog with an open left ring and an isoleucine replacing the naturally occurring di-hydroxy-isoleucine as amino acid 3 (aa3) of α-amanitin, was free of inhibitory activity despite the high concentrations tested. The synthetic α-amanitin analogs HDP30.0528 (Figure 1D), containing an open left ring and serine instead of the di-hydroxy-isoleucine as aa3, as well as HDP30.0470 (Figure 1E), an isoleucine-amanitin analog with closed left ring showed at both tested concentrations an inhibitory activity that was comparable with the activity of α-amanitin at the 10–100 - fold lower concentrations. These results demonstrated that the assay established so far allowed for a qualitative assessment of the inhibitory activity. However, smaller differences in the potency of various inhibitors could not be determined.

Attempts for qPCR optimization including the use of an alternative primer pair, EGFP-frw2 and EGFP-rev2 amplifying a shorter PCR fragment (Table 1), did not lead to any significant improvement. Since the EGFP template was not suited for more effective primer design, the positive control DNA provided in the HeLaScribe kit was chosen as an alternative. Primers were designed to amplify the HS-DNA linear fragment containing the CMV promoter and the transcribed sequence (Table 1 and Additional file 1). Several different primers pairs and two sequence specific probes were designed within the transcribed sequence (Table 1). Upon optimization, the primer-probe mix H1 gave the best qPCR results.

### Template immobilization and optimized qPCR quantification

To remove the DNA after transcription, the two biotinylated linear DNA templates CMV-EGFP and HS-DNA were synthesized and immobilized on magnetic beads. qPCR analysis of the supernatant, however, showed that the DNA interference was still considerable (Table 2 and Additional file 2: Figure S1A). The RNA purification step could thus not be avoided by using the immobilized template. Since the bead-bound HS-DNA was most efficiently transcribed, even when compared to the free linear one (see Additional file 2: Figure S1B), it was chosen as a template for all subsequent experiments.

**Table 2:** Template comparison

**Template**		**qPCR C(T) values**		**qPCR C(T) values**	
		**No RNA purification**		**RNA purification**	
		**Positive**	**Negative**	**Positive**	**Negative**
CMV-EGFP	Free	32.6	31.6	17.4	>40
	Beads	37.3	>40	23.2	>40
HS-DNA	Free	27.7	22.3	30.3	37.0
	Beads	28.1	29.6	20.7	>40

Two different linear DNA templates, CMV-EGFP or HS-DNA in solution (free) or immobilized on magnetic beads (beads) were used. Only positive transcription (+) and negative control reactions (−) were performed, and the product detected using primer extension and qPCR. C(T) values for the positive reactions should be lower than 25, values over 30 for the positive reactions reflect RNA synthesis levels that are too low to allow reliable inhibition quantification. Negative reactions should result in C(T) values higher than 35. C(T) values > 40 reflect no amplification, i.e. no detectable RNA synthesis.

**Figure 4:**
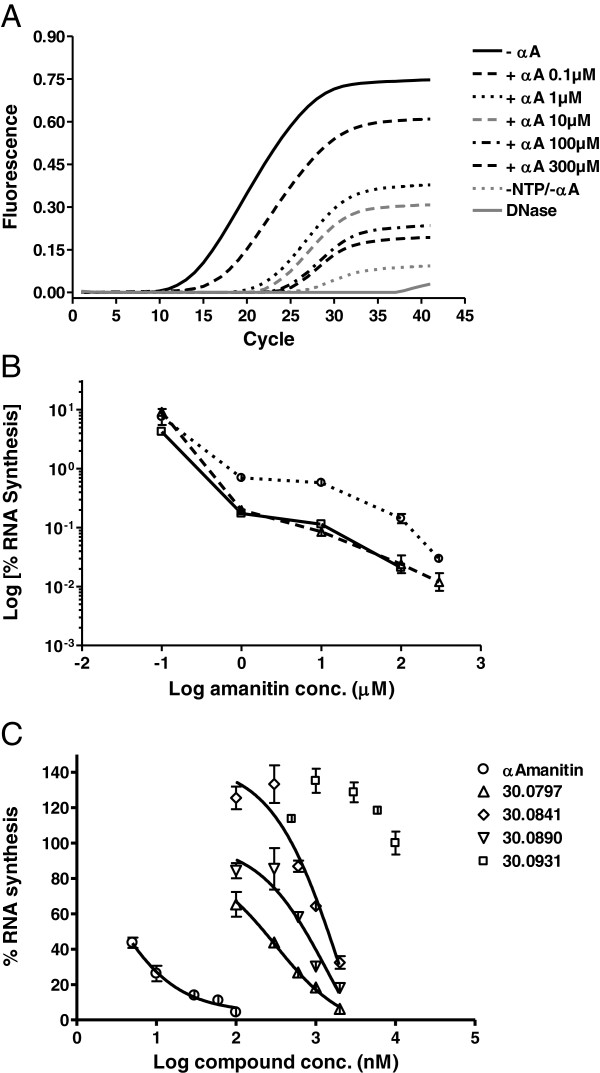
**Quantitative analysis of RNA synthesis by optimized qPCR.***In vitro* transcription was performed using the linear, bead immobilized DNA template Bead-HS-DNA. Each reaction was performed in duplicate. The RNA was purified, DNase digested and analyzed by reverse transcription followed by qPCR amplification. **A**. The amplification curves of RNA products derived from uninhibited and α-amanitin-containing reactions showed gradually decreasing C(T) values with increasing α-amanitin concentrations. For each type of reaction, the average fluorescence values were plotted. -αA – transcription reactions without α-amanitin; +αA - transcription reactions in the presence of α-amanitin; −NTP/-αA – negative control transcription reactions. **B**. Graphic representation of the calculated percent RNA synthesis in the presence of amanitin derived from the qPCR curves presented in **A** and from two additional similar experiments. Due to the high amanitin concentrations used, the residual percent RNA synthesis was lower than 10% and is depicted in a logarithmic scale. **C**. The optimized method was used to compare the inhibitory activity of amanitin to that of four synthetic analogs. **B** &**C**. The newly synthesized RNA amount was calculated using a DNA standard curve. By relating to the RNA amount synthesized in the uninhibited reactions a residual percent RNA synthesis was calculated. Bars show standard deviation.

A series of transcription and qPCR optimization experiments resulted in an optimized method for the *in vitro* RNA polymerase assay, which is detailed in the Additional file 3. This method was applied to assess the inhibitory activity of α-amanitin in several independent experiments. The quantification of the synthesized RNA showed a clear α-amanitin-concentration dependency (Figure 4A & B) and a very good reproducibility over several experiments (Figure 4B).

When applied to assess the inhibitory activity of several α-amanitin analogs vs. α-amanitin, this optimized method allowed for a quantitative estimation of the inhibitory potency using the inhibition concentration 50 (IC_50_) values (Figure 4C). Three of the tested synthetic α-amanitin analogs HDP30.797 (Figure 1E, IC_50_ = 314 μM), HDP30.841 (Figure 1E, IC_50_ = 2.1 mM) and HDP30.890 (Figure 1E, IC_50_ = 1.4 mM), sharing the α-amanitin structure but with the natural amino acid 3 (aa3) di-hydroxy-isoleucine replaced by different synthetic phenyl-glycine derivatives, showed 150-1000-fold weaker inhibitory activity than α-amanitin (IC_50_ = 2.1 μM). The α-amanitin-analog HDP30.0931 (Figure 1E), an aa3 α,α-dihydroxy-methyl-glycine derivative, showed no inhibitory activity up to concentrations as high as 10 mM (IC_50_ > 10 mM).

### RNA polymerase assay with Quantigene quantification

The optimized qPCR detection method allowed for very precise and specific RNA quantification after transcription. However, due to the indispensable RNA purification step, the assay remained very laborious. The Affimetrics QuantiGene assay, described as a qPCR alternative, which still allows detection of minute RNA amounts but does not require RNA purification, was considered and tested. For a first attempt, RNA was transcribed by the optimized method in positive and negative (no NTPs) *in vitro* transcription reactions. After transcription, half of the reaction mixture was used for RNA purification and the synthesized RNA amount was quantified using the optimized qPCR method. The unpurified positive reaction mixture and the purified RNA derived from it were then serially diluted and assessed by the QuantiGene method.

**Figure 5:**
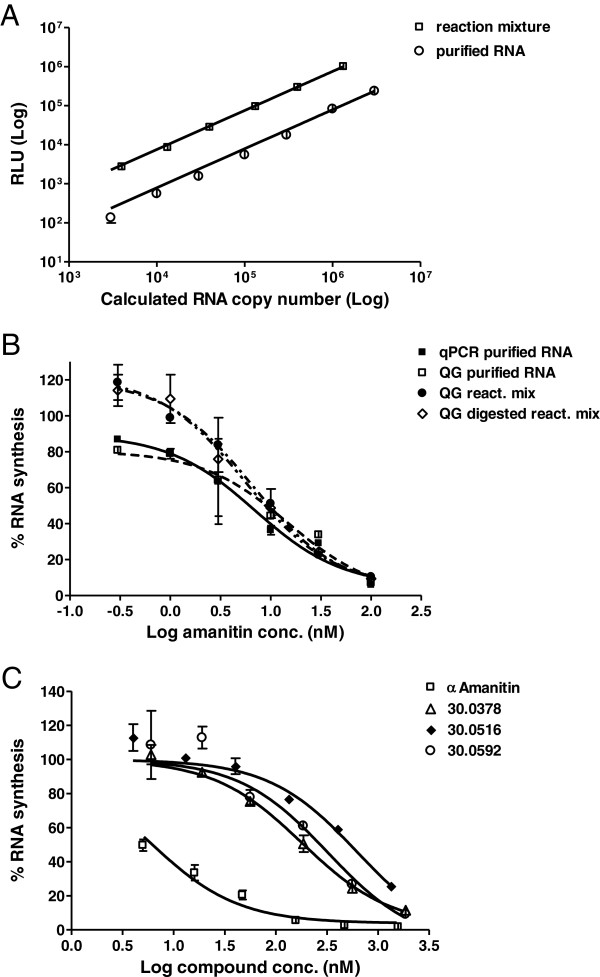
**Quantitative analysis of RNA synthesis by QuantiGene method. A** &**B**: *In vitro* transcription reactions were performed using the linear, bead immobilized DNA template Bead-HS-DNA. Half of each reaction mixture was subsequently used for RNA cleanup and the RNA amount was analyzed by the optimized qPCR method. **A**. Dilutions were either prepared directly from the reaction mixture or from the purified RNA. Each dilution was assessed as a 4-fold replicate by the QG method. The chemoluminescence signals were plotted against the expected RNA copy number, showing a clear linear relationship. Error bars show standard deviation. **B**. The optimized qPCR method was used to quantitate RNA inhibition by α-amanitin as described in Figure 4. In parallel, purified RNA, diluted reaction mixtures or DNase-digested reaction mixture dilutions were analyzed by the QG-method. Percent RNA synthesis was calculated by relating the luminescence signals of the respective α-amanitin-reaction to the signal of the uninhibited reaction. The QG method provided clear α-amanitin-concentration dependency curves using unpurified RNAs directly from the reaction mixture. **C**. The QG-method was applied to compare the inhibitory activity of α-amanitin to that of three semisynthetic derivatives. Bars show the standard deviation of the duplicate samples.

A clear RNA concentration dependency of the signal was seen for both the purified RNA and the unpurified reaction mixture dilutions (Figure 5A). Moreover, the signal showed a clear linear relationship to the degree of dilution over three orders of magnitude. The purified RNA from the negative control showed as expected only a background-level signal, but a significant signal, probably derived from DNA interference, was observed in the negative control when the negative reaction mixture was assessed without prior RNA purification. However, this signal (236,494 RLU), derived from an 1:2 diluted reaction mixture was <20% when compared to the signal of the 1:10 dilution of the reaction mix (1,991,564).

A second experiment was set up to compare the QuantiGene and qPCR detection of *in vitro* transcribed RNA from a set of reactions containing serial α-amanitin dilutions. For qPCR quantification, half of the reaction mix was subject to RNA purification. Three different series were used to the QuantiGene detection: an aliquot of the purified RNA, a dilution prepared directly from the reaction mix and the same reaction mix dilution that was digested by DNase prior QuantiGene assessment. As expected, almost identical inhibition curves were obtained when the purified RNA was assessed by the QuantiGene or qPCR methods. Most importantly, the inhibition curves derived from the reaction mix samples also showed a clear concentration dependency and did not differ significantly from the curves achieved using the purified RNA, regardless of the DNase digestion step (Figure 5B). The QuantiGene RNA quantification method therefore proved to be a worthy alternative for the reverse transcription plus qPCR, combining its sensitivity with the huge benefit of avoiding RNA purification.

This method was thus applied to compare the inhibitory activity of α-amanitin and α-amanitin-derivatives in a series of *in vitro* transcription reactions (Figure 5C). In this experiment, three semisynthetic amanitin-linker derivatives were assessed, all of them being methylated on the hydroxyl-group of amino acid 4, 6-hydroxy-tryptophan, and containing the linker bound to the N1 of the same amino acid. These compounds only differed in the linker length, which varied between four, six and eight carbon atoms in HDP30.0592, HDP30.0378 and HDP30.0516 (Figure 1F), respectively. The IC_50_ values of the three compounds, HDP30.0592 (IC_50_ = 321 μM), HDP30.0378 (IC_50_ = 180 μM) and HDP30.0516 (IC_50_ = 629 μM), were 30-100-fold lower than of α-amanitin (IC_50_ = 5.7 μM), demonstrating that all these compounds could still bind to the RNA polymerase II despite methylation and substitution by linker, and that an optimum of activity could be achieved when using a linker with a length of 6C atoms.

### Effect of TATA-binding protein (TBP) on the transcription efficiency

To assess for transcription efficiency in the absence of TBP activity, a template lacking the TATA box was used, in which the TATATA sequence had been substituted by the TAGCTA sequence (Additional file 4). Positive, negative and 200 nM α-amanitin-containing transcription reactions were set up as duplicates using either the standard bead-immobilized HS-DNA template or the same template containing the TATA box mutation. After transcription and sedimentation of the beads, an aliquot of each reaction mix was analyzed by the QuantiGene assay as described in the Additional file 3. Quantification of the newly synthesized RNA products revealed that an amanitin-sensitive transcription occurred on both templates (Table 3). 200 nM α-amanitin inhibited the transcription efficacy by 88 ± 1% for both templates. When compared to the standard template, transcription efficacy from the template with mutated TATA box was 20-30% lower.

**Table 3:** Effect of TATA-binding protein on the transcription efficiency

**Template**	**Reaction**	**Signal (RLU average)**	% RNA synthesis*	**% RNA synthesis**
				TATA mutant vs. standard**
HS-DNA_std	Positive	1306740	100% ± 3%	100% ± 3%
	Negative	9768		
	α-amanitin 200 nM	161585	12% ± 1%	
HS-DNA_mut	Positive	985650	100% ± 10%	75% ± 7%
	Negative	11795		
	α-amanitin 200 nM	130255	12% ± 1%	

Transcription reactions were performed using the standard DNA template (HS-DNA_std) or a TATA-box mutated template (HS-DNA_mut). Transcription efficacy was determined by QuantiGene quantification.

*Compared to respective average positive control, less the respective negative controls.

**Compared to average positive control on standard template, less the respective negative controls.

### qPCR and QuantiGene quantification of the inhibitory effects of flavopiridol

Both the optimized qPCR detection method as well as the QuantiGene method were applied to assess the flavopiridol effect on the transcription efficacy. Transcription reactions were performed and analyzed as described in the detailed protocol in the Additional file 3. Flavopiridol was added in concentrations ranging from 200 nM to 625 mM, parallel control reactions contained 64 pM to 200 nM α-amanitin. The first set of reactions was performed using the standard bead-immobilized HS-DNA template and the transcription reactions were incubated for 30 min at 30°C. A second set of reactions was performed using the longer template HS-DNA_long sharing the same promoter but rendering a 98 bp longer transcript (456 vs. 358 bp) i.e. a 98 bp longer primer extension and qPCR detection fragment (Additional file 4). This set of reactions was incubated for transcription for a shorter period of 10 min (vs. 30 min for standard conditions) at 30°C.

**Figure 6:**
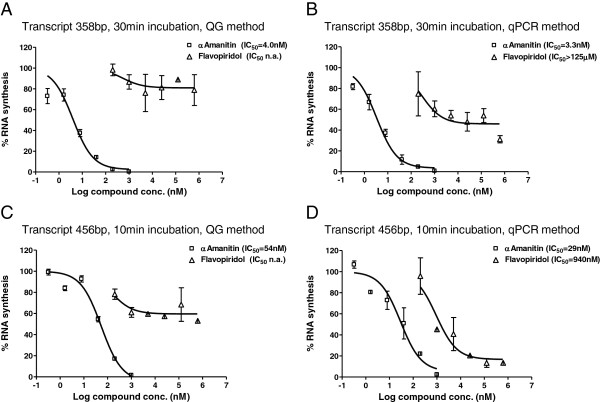
**Comparison of the QuantiGene and qPCR methods as used for assessing the inhibitory effect of flavopiridol. A** &**B**. Transcription reactions in the presence of α-amanitin or flavopiridol were set up using the standard DNA template HS-DNA and performed following the standardized protocol as described in the Additional file 3. **C** &**D**. A 98 pb longer DNA template, HS-DNA_long, was used to set up transcription reactions in the presence of α-amanitin or flavopiridol. Reaction conditions were similar to the experiment in **A** &**B** except for a shorter incubation period of 10 min instead of 30 min in the standard setting. To quantitate the RNA synthesis yield, the optimized, standardized QG- **(A & C)** or qPCR-method **(B & D)** was applied. Residual percent RNA synthesis was calculated by relating to the RNA amount in the positive control reactions. Bars show the standard deviation of the duplicate samples.

In contrast to α-amanitin which showed similar inhibition effects regardless of the method used for quantification, the measured inhibitory effects of flavopiridol on the RNA synthesis depended on the quantification method. Generally, the QuantiGene method resulted in lower inhibition data (Figure 6A & C), while figures generated by the primer extension & qPCR method indicated higher inhibitory effects (Figure 6B & D). Moreover, considering the figures from the qPCR quantification method, a higher degree on inhibition was measured for flavopiridol in the second set of reactions generating the longer transcripts within a shorter incubation period (IC_50_ = 940 nM, Figure 6B), when compared to the first set (IC_50_ > 125 μM Figure 6D). In contrast, the inhibitory effect of amanitin was ca. 10× lower for the reactions with short incubation periods.

## Discussion

Eukaryotic transcription is a highly complex and tightly regulated process and therefore extremely difficult to reconstitute *in vitro*. It is commonly divided into the six steps preinitiation, initiation, promoter escape/clearance, elongation, and termination, all being the subject of tight regulation. Except for the termination, an *in vitro* run-off transcription system must emulate a proper environment for all these processes [15]. Transcription initiation depends on the correct assembly of an active pre-initiation complex at the site of the promoter. In the past years, many different RNA polymerase II promoters with different DNA sequences were described, requiring multiple types of pre-initiation complexes to be formed [16]. After initiation, RNA polymerase II starts RNA synthesis by successively adding nucleotides to a growing RNA chain. This “promoter clearance” [17,18] marks the transition from initiation to elongation which is a highly dynamic and tightly regulated stage of the eukaryotic transcription, too [19,20]. Minimal requirements for RNA polymerase II and cofactors for activity at specific promoters are now known and highly purified transcription systems have been described [21]. One commercially available transcription system is the HeLaScribe Nuclear Extract that has been used for the studies described in this paper.

However, it is not trivial to perform an *in vitro* transcription assays even when using a kit. In our hands, its main limitation proved to be its ineffective initiation and low rate of transcription that had been also described by others [15]. The RNA amounts synthesized in one reaction varied around 1×10^8^ copies; the detection of such small amounts in the presence of a 1000-fold excess DNA template turned out to be very challenging. Moreover, for comparing various RNA polymerase II inhibitors, a precise quantification of the synthesized RNA was required. Gel electrophoresis and autography, as normally used for RNA detection in the field of transcription research, were not considered to fit for our purpose.

The pEGFP-N1 plasmid was selected as a template, since it had worked in our hands as an RNA polymerase II template in cells upon transfection. For detection, the primer extension method was chosen and complemented by a PCR step for signal amplification. The main challenge of this system was to ensure that DNA contaminations would not interfere with the RNA quantification. Two negative controls were designed to control the DNA removal: the ‘negative control transcription’ reactions that contained the complete reaction mix including the DNA but no NTPs, as well as a ‘DNase control’, a DNA sample that was directly digested and then subject to reverse transcription and PCR as the purified RNA samples.

The results of these experiments were highly encouraging, since the PCR method clearly detected newly synthesized RNA and α-amanitin showed a concentration-dependend effect on the detected RNA amount. This concentration-dependency, however, could not be translated into numbers by switching to a qPCR detection system, though the assay itself proved to be strikingly robust and reproducible. Since there was no room for PCR optimization within the EGFP translated sequence, another DNA template had to be selected and the positive control DNA from the HeLaScribe kit, the HS-DNA, was chosen. For this template, a very efficient, sequence-specific probe dependent qPCR detection method was established prior to proceeding with the further optimization of the transcription assay.

In parallel, magnetic bead immobilization of the linear DNA fragments was considered to avoid the very laborious RNA purification step by fishing out the DNA from the mixture. However, DNA contamination of the bead supernatant exceeded the RNA amount and hindered its direct quantification. On the other hand, transcription from the immobilized HS-DNA template was very efficient, so that the immobilization step was kept for the optimized protocol. Interestingly, transcription from the immobilized CMV-DNA was very poor. This probably is due to the fact that the DNA stretch 5′ of the promoter is much shorter in the CMV-EGFP DNA than in the HS-DNA. Immobilization of the CMV-DNA template probably results in a steric hindrance of the transcription initiation step.

Finally, all steps of the transcription assay were optimized for maximal efficiency and minimal complexity. The transcription reaction volume was reduced back to 25 μl and reactions were performed as duplicates. For the RNA purification, the RNeasy Micro kit was replaced with the more convenient RNeasy Mini kit, which is also available as a 96-well format. Freeze-drying the RNAs overnight in a speedvac compensated the higher elution volumes. The dried RNAs were dissolved directly into the DNase digestion buffer. The volume of the digestion mixture that could be used for reverse transcription was maximized and the reactions were set up in qPCR vessels, so that the qPCR master mix aliquots could be subsequently directly added. The qPCR parameters temperature, concentration of primers, probe and Mg^2+^ were optimized for highest reaction efficiency.

This optimized method was ready to be applied to quantitatively assess the inhibition profile of RNA polymerase II inhibitors. However, its requirement for RNA purification limited the number of samples that could be processed simultaneously. We therefore continued searching for detection methods that would allow skipping the purification step and identified the QuantiGene assay as an alternative. The use of hybridization for the RNA capture step renders this method very specific, while multiple subsequent hybridization steps and an enzymatic reaction achieve a high degree of amplification and allow detection of very low amounts. We thus tested this method for the quantitation of RNA directly in the transcription mixture. Although some DNA interference in the negative controls leading to slightly positive detection results could not be avoided, the dynamic range and linearity of the assay were compelling. A direct comparison of the results of the optimized qPCR with the QuantiGene method finally demonstrated that the latter provided similar results without a need for RNA purification or DNase digestion, thus considerably simplifying and speeding up the assay. Using the QuantiGene assay, eukaryotic transcription assays can be performed in a 96-well format and eventually fully automatized, so that a high number of inhibitors can be concomitantly analyzed.

Beside its simplicity, the main advantage of this new method is the precise quantification of the transcription reaction yield. For example, α-amanitin-derivatives modified by the same linker chemistry that differed only in the linker length showed distinct inhibition profiles with the longest linker showing slightly less potency than the shorter ones. This finding could be reproduced in cells in cytotoxicity experiments, after the respective amanitin-linker derivatives had been coupled to an IgG targeting an antigen on the cells (data not shown).

In another set of experiments, the activity of flavopiridol was assessed, an alternative inhibitor of transcription elongation. Flavopiridol acts on transcription by stoichiometric binding to P-TEFb, a protein kinase composed of the cyclin-dependent kinase 9 (cdk9) and a cyclin subunit, which regulates transcription elongation by phosphorylating the C-terminal domain (CTD) of the large subunit of the RNA polymerase II [22,23]. As expected, the measured inhibitory effect of flavopiridol on the standard template was significantly lower compared to α-amanitin. α-Amanitin directly binds with very high affinity to the polymerase core subunit and blocks its translocation to the next nucleotide [24], its IC_50_ inhibition values thus depend on the concentration of RNA polymerase core subunit. On the other side, by binding to P-TEFb, flavopiridol inhibits the phosphorylation of the RNA polymerase core subunit. Slower transcription elongation at 20–50 nucleotides per min, however, occurs even in the absence of CTD phosphorylation and the resulting transcripts are therefore shorter [25]. The quantification results reflected these features of flavopiridol inhibition. A higher residual RNA synthesis rate was measured by the QG method than by qPCR, due to the synthesis of short RNA molecules that are missing the reverse priming site used for primer extension and qPCR. As expected, when a longer template was used and the incubation time shortened, a significantly higher inhibition degree was assessed for flavopiridol using the qPCR method. However, more thorough investigations would be required to explain the varied results from the flavopiridol experiments.

But apart from a high-throughput assessment of transcription inhibitors, the new methods can be employed for a large variety of transcription-related applications, since the transcript sequence can be combined with any desirable promoter. As an example, a DNA template containing a mutated TATA box was used for *in vitro* transcription reactions in comparison to the wild-type template. Using the QuantiGene quantification method a transcription yield of ca. 75% of the wilde-type was observed for the TATA mutant template. An intact TATA box determines the transcription start position by providing the binding site for TBP but is not strictly required for human transcription to occur. The mutation can cause a shift of the start of transcription site to one or multiple weaker ones, as well as influence the transcription efficacy [26]. Our results suggest that the HeLa Scribe nuclear extract used contained several transcription initiation factors that compensate for the absence of TBP binding and activate alternative pre-initiation pathways.

Moreover, provided that the composition of the transcripts has been previously analyzed to ensure an accurate interpretation of the results, the QuantiGene detection can be easily adapted for the detection and quantification of any specific transcript. The transcription reaction composition can be changed so that the effect of specific factors can be independently and quantitatively assessed. By using immobilized DNA templates, pre-initiation complexes can be assembled, isolated and their translational activity further investigated. The immobilized linear DNA can be processed to reconstitute a more physiological nucleosomal template for *in vitro* transcription experiments, since the ultrasensitive RNA detection compensates for the low initiation efficacy of such *in vitro* transcription assays. However, determining the composition of the transcripts would be required before determining potential HTS set-up.

## Conclusions

In conclusion, on the basis of a commercially available kit we have established a relatively simple, robust and reproducible non-radioactive method for RNA quantification after eukaryotic *in vitro* transcription. We showed that a simple qualitative PCR detection of the RNA transcript can be used after a run-off assay to avoid radioactive labeling. Moreover, the optimized qPCR detection method allows for unprecedented precise quantification of the transcription efficacy. When using the QuantiGene detection, the laborious RNA purification can be avoided and a large number of samples processed simultaneously.

## Methods

### Linear template amplification and immobilization; oligonucleotide design

The pEGFP-N1 plasmid (Clontech, sequence accession no. U55762) was used as a template for the amplification of the 1136 bp linear DNA fragment CMV-EGFP using the *CMV-EGFP-frw* and *CMV-EGFP-rev* primers. The positive control DNA (for sequence see Additional file 1) from the HeLaScribe Nuclear Extract *in vitro* Transcription System (Promega) was used as a template for the PCR-amplification of the 1182 bp linear DNA fragment HS-DNA using the *HELA NUCLEAR FW* and *HELA NUCLEAR RV* primers (for the sequences of all used primers see Table 1). The same primers were used to amplify the alternative linear fragments HS-DNA_mut and HS-DNA_long containing either an intact promoter but a mutated TATA box and the same transcripted region, or a 98 bp longer transcripted region with intact promoter and TATA box (see Additional file 4). Amplified linear DNA fragments were purified using the Qiagen QIAquick PCR Purification Kit. This method does not remove the template DNA from the mixture, which results in some plasmid contamination of the linear template. However, gel-purified DNA did not work for *in vitro* transcription, probably because of the dye interfering with the transcription machinery. For immobilization of the linear templates onto Dynabeads M280 (Life Technologies), the respective 5′-biotinilated forward primers were used. Bead immobilization was performed as recommended by the manufacturer at 0.5 μg DNA per 100 μg beads and stored at 4°C in the binding buffer. The binding efficacy of about 80% resulted in a DNA concentration of 0.4 ng/100 g beads. Prior to transcription reaction setup, the required amount of loaded beads was transferred to a new tube and washed there times with *in vitro* transcription buffer.

Two primer pairs were designed for PCR and qPCR detection on the CMV-EGFP – derived RNA/cDNA, six for the detection of the HS-DNA transcription product, of which only the two sets showing satisfactory results are shown in Table 1. For reverse transcription, the reverse primer closest to the transcript’s 5′ end was used, i.e. *EGFPrev1* for CMV-EGFP and *HNqPCRrev1* for HS-DNA. For Quantigene detection, the customized oligonucleotide mix designed by the manufacturer based on the sequence of the transcribed region of the HS-DNA template.

### Natural, synthetic and semisynthetic α-amanitin derivatives; other chemicals

Flavopiridol was obtained from Sigma-Aldrich. α-Amanitin (Figure 1A) was obtained from AppliChem. The semisynthetic amanitin derivative 6′–O-methyl-γ-amanitin (Figure 1C) was a gift of Prof. H. Faulstich [14].

The semisynthetic linker derivatives HDP30.0378, HDP 30.0516 and HDP 30.0592 (Figure 1F), derived from natural α-amanitin, were obtained by methylation of the phenolic group of the 6-hydroxytryptophan moiety and subsequent alkylation of the indol nitrogen (N1) of 6-O-methyl-α-amanitin using protected bromoalkanamines with different carbon lengths. Methylation was required to block the more acidic OH group. The (N1)–H deprotonation reaction was performed using LiOH or potassium-t-butoxide.

HDP30.0346 (Figure 1B) was synthesized by solid phase peptide synthesis according to standard peptide synthesis protocols. Thus Rink amide resin was used followed by Fmoc peptide coupling. Key step was the integration of a cysteine-tryptophan precursor as described in literature [27]. The pentamer was subsequently de-protected and cleaved off from resin. The ring was finally cyclized in solution by use of standard coupling reagents under high dilution.

The synthetic derivatives HDP30.0445, 30.0528, 30.0470, 30.0797, 30.0841, 30.0890 and 30.0931 (Figure 1D & E) were synthesized using standard peptide synthesis protocols. The procedure for the transformation of the respective linear octapeptides into the amanitin bicyclic structure was based on the “Savige Fontana” technique [28]. The monocyclic synthesis precursors HDP30.0445 and HDP30.0528 (Figure 1D) contain an open left side.

### *In vitro* transcription assay followed by primer extension and PCR/qPCR detection

Transcription reactions were set up as using the Promega HeLaScribe Nuclear Extract *in vitro* Transcription System were set up as described by the manufacturer with some modifications. The reaction volume was doubled to 50 μl. A master mix was prepared from a volume transcription buffer (20 mM HEPES, pH 7.9, 100 mM KCl, 0.2 mM EDTA, 0.5 mM DTT, 20% glycerol) corresponding to 22 μl minus the nuclear extract volume, MgCl_2_ to a final concentration of 3 mM, 40 Units RNase inhibitor, DNA template (1 μg pEGFP-N1 plasmid or 150 ng linear DNA fragment CMV-EGFP, respectively) and the nuclear extract following the manufacturer’s recipe. Aliquots of the master mix were transferred into reaction tubes containing different α-amanitin concentrations or water and incubated for 20 min at room temperature. Standard transcription reactions containing no α-amanitin were referred as ‘positive’, reactions containing α-amanitin or other inhibitors as ’inhibited’. A standard reaction containing no α-amanitin/inhibitors but also no NTPs was used as a ‘negative control transcription’. The reactions were started by the addition of the NTP mix to a final concentration of 400 μM each, except for the negative control where water was added. After incubating for 30 min at 30°C, the reactions were stopped by diluting with 400 μl of the RLT buffer from the Qiagen RNeasy Micro kit and stored at −80°C until proceeding with RNeasy cleanup. This was performed as recommended by the manufacturer, including on-column DNase treatment but without the addition of carrier RNA. The RNA was eluted using 15 μl water and stored at −80°C.

The purified RNA was once more DNase-treated using the Epicentre BaselineZero DNase kit as recommended by the manufacturer, 8.8 μl of the RNA being digested in a final volume of 10 μl. As a second negative control, 50 ng of the linear DNA fragment CMV-EGFP were directly DNase-digested and further processed as the RNA samples (‘DNase control’). For reverse transcription with the Qiagen Sensiscript RT kit, 2 μl of the digestion reaction and 1 μM *EGFPrev1* primer (Table 1) were used in a final reaction volume of 20 μl. After reaction, 1 μl of the reverse transcription mix was transferred to PCR tubes containing 24 μl PCR mix aliquots. The standard PCR amplification was performed using the Epicentre Failsafe PCR system with the Mix A and the *EGFPrev1* and *EGFPfrw1* (Table 1) primer mix. Positive PCR control reactions contained 50 ng pEGFP-N1 plasmid; negative PCR controls contained no DNA. After amplification, the products were analyzed by gel electrophoresis onto 1.5% agarose.

For quantitation using SYBR detection, the Qiagen QuantiFast SYBR green qPCR kit was used. 1 μl of the reverse transcription mix was amplified in 25 μl reaction mix containing 500 nM of each EGFP-rev1 and EGFP-frw1 primer. For quantitation using the sequence-specific LNA probe *EGFP-LNA1* at 250 nM, same primer set as well as an alternative primer set (*EGFPfrw2* and *EGFPrew2*, Table 1) at 500 nM each and Sigma Jumpstart Taq ReadyMix for qPCR were used. 2 μl of the reverse transcription reaction were diluted into qPCR reactions with a final volume of 20 μl and qPCR performed in a Bio-Rad DNAEngine lightcycler. A qPCR standard curve was prepared from the CMV-EGFP DNA fragment and used for RNA copy number calculations. The amount of RNA in the inhibited probes was related to the RNA amount detected for the positive transcription reaction. A particular compound concentration thus shows an inhibitory effect if its ration is < 1.

### *In vitro* transcription assay with immobilized templates followed by qPCR quantification

Duplicate transcription reactions using immobilized template were set up in 25 μl the same way as the reactions with linear templates, except that the transcription buffer was replaced by the freshly prepared DNA-bead suspension in transcription buffer. The template amount was set to 150 ng immobilized DNA per 50 μl reaction. The required buffer volume and thus the bead concentration were calculated considering the activity of the nuclear extract charge used, as described in the HeLaScribe manual. The master mix containing the bead suspension in transcription buffer, 3 mM MgCl_2_ and 40 Units RNase inhibitor was supplemented with the HeLa nuclear extract, mixed by gentle vortexing and dispersed in aliquots into the reaction tubes. Water or α-amanitin in various concentrations was added to the tubes, mixed gently and incubated for 20 min at room temperature. Reactions were started by the addition of the NTPs, except for the negative control transcription reaction, where water was added. During the 30 min incubation at 30°C the reaction tubes were twice gently vortexed to resuspend the beads.

After reaction, the beads were spun down and 20 μl of the supernatants were diluted into the RLT buffer of the Qiagen RNeasy Mini kit and stored at −80°C until proceeding to the RNA cleanup. The RNA was eluted from the RNeasy mini-columns with 2×30 μl water and subsequently dried using a speed-vac. DNase digestion and reverse transcription steps were performed as described above with some modifications. The dried RNA was directly taken up into 10 μl DNase digestion mix. For reverse transcription in a final volume of 10 μl using 3 μl of the DNase-digested RNA, the *EGFP-rev1* (Bead-CMV-EGFP template) or *HNrev1* (Bead-HS-DNA template) primers were used. After reverse transcription, 15 μl qPCR master mix aliquots were added directly to the reverse transcription mixtures and the amplification was performed as described. For the Bead-CMV-EGFP - derived RNA the primer-probe-mix EGFP1 (final concentrations *EGFP-frw1* 500 nM, *EGFP-rev1* 500 nM, *EGFP-LNA1 2*50 nM) was used, for the Bead-HS-DNA - derived RNA the primer-probe-mix H1 (final concentrations *HNfrw1* 500 nM, *HNrev1* 2 μM, *HN_SONDE1* 250 nM). A qPCR standard curve was prepared from the respective template DNA and used to calculate the RNA copy number in the samples. By relating the RNA copy numbers in the inhibited reactions to the copy number in the positive transcription reactions, a percent transcription inhibition was calculated for each compound and concentration and an inhibition curve plotted. A sigmoidal dose–response curve fit was used to calculate the compound concentrations that inhibited RNA synthesis by 50% (inhibiting concentration 50, IC_50_).

### RNA polymerase assay with QuantiGene quantification

Positive and negative control transcription reactions using the bead-immobilized Bead-HS-DNA template were set up as duplicates as described above in a final volume of 100 μl per reaction. After transcription, 50 μl of the reaction mixture were used for RNA cleanup. The copy number of synthesized RNA was quantitated using the established primer extension and qPCR method. From this number, the RNA copy number in the reaction mixture was estimated. Using the rest of the reaction mixture of the positive reactions, a dilution series was prepared starting at 1:10 unto 1:10,000. 0.3 mM EDTA was added to the first dilution to bind Mg^2+^ that would interfere with the QuantiGene capture step. Alternatively, a dilution series was prepared from the purified RNA, starting at 1:100 unto 1:100,000. Both dilution series were assessed using the QuantiGene 2.0 System (Affimetrix) following the instructions provided by the manufacturer. The negative control transcription reaction products were only assessed as a 1:2 dilution for the unpurified negative reaction mix or 1: 10 dilution for the DNase-digested RNA purified from the negative control transcription reaction.

For the capture step, the working reagent was prepared according to the recipe provided in the ‘Capturing Target RNA from Fresh, Frozen, or FFPE Tissue Homogenates’ section of the QuantiGene Handbook. 60 μl of working reagent were distributed into the QuantiGene plate and 40 μl of reaction mix or RNA dilution were added. The subsequent hybridization, signal amplification and detection steps were performed as described in the manual. Finally, the chemoluminescence signals were measured using a FLUOstar Optima chemoluminometer (BMG LABtech) and were plotted against the estimated RNA copy number.

For further experiments, positive, inhibited and negative control transcription reactions were set up in 25 μl as duplicates covering a complete range of α-amanitin or compound concentrations. After transcription, 20 μl of the bead supernatant was used for RNA cleanup followed by the optimized primer extension and qPCR quantification method. 3 μl of the reaction mix were diluted with 97 μl water. 4 μl of this dilution were once more diluted with 45 μl 0.3 mM EDTA containing RNase-inhibitor (2U/μl). Alternatively, 36 μl DNase Zero master mix containing buffer, RNase inhibitor and enzyme were added to 4 μl of the reaction mix dilution and DNase digestion was performed for 15 min at 37°C. The digestion was stopped by the addition of 5 μl stop solution (EDTA) and 10 min heating at 65°C. A blank DNase digestion with water was used for the Qiagen background sample. For QuantiGene quantification, 40 μl of the purified RNA, the twice diluted reaction mix or the digested diluted reaction mix were added to 60 μl working reagent in the QuantiGene capture plate. Upon chemoluminescence detection, the background signals were subtracted from the signals of the positive or inhibited or negative control transcription samples. For each sample type, reaction mixture, digested reaction mixture and purified RNA, the negative control sample signals were averaged and subtracted from the signals of the respective positive and inhibited samples. A percent inhibition was calculated by relating the signals of the inhibited reactions to the signal derived from the positive transcription reactions, which were set to 100%. A sigmoidal dose–response curve fit of the inhibition curves was used to calculate the IC_50_ values of the tested compounds.

## Authors’ contributions

CV and JA developed the concept and co-wrote the manuscript; CV and BS performed most experiments; SW-S, CL and WS performed the chemical synthesis of the derivatives; JA supervised the project. All authors read and approved the final manuscript.

## Supplementary Material

Additional file 1 **HeLaScribe Positive Control DNA Sequence.** DNA sequence information for the DNA template ‘HeLa Nuclear Extract Positive Control DNA’. Click here for file

Additional file 2 **Template comparison.** qPCR amplification curves of the data presented in Table 2. Click here for file

Additional file 3 **Detailed Protocol.** Detailed protocol for non-radioactive eukaryotic *in vitro* transcription and RNA quantification. Click here for file

Additional file 4 **Alignment of the HS-DNA, HS-DNA_mut and HS-DNA_long sequences.** HS-DNA_mut is a mutant of the standard HS-DNA template containing a mutated TATA box. HS-DNA_long is a template similar to the standard HS-DNA template but including additional 98 bp within the run-off transcript (and primer extension/qPCR product) sequence. The sequence alignment highlights the differences between the three templates. Click here for file
