# Supplementary material for: A novel, non-radioactive eukaryotic in vitro transcription assay for sensitive quantification of RNA polymerase II activity
Source: BMC Mol Biol. 2014 Apr 3;15:7. doi: 10.1186/1471-2199-15-7 (PMC4021065; doi:10.1186/1471-2199-15-7)
Supplement: Additional file 1 — HeLaScribe Positive Control DNA Sequence. DNA sequence information for the DNA template ‘HeLa Nuclear Extract Positive Control DNA’. [file 1471-2199-15-7-S1.docx]

Nucleotide Sequence

of the ‘HeLa Nuclear Extract Positive Control DNA (CMV)’ from the ‘HeLaScribe® Nuclear Extract in vitro Transcription System’ as provided by the manufacturer (Promega).

1 gtgctgcaag gcgattaagt tgggtaacgc cagggttttc ccagtcacga cgttgtaaaa

61 cgacggccag tgaattctca tgtttgacag cttatcgatc cgggcaacgt tgttgccatt

121 gtcgcaggcg gagaactggt aggtatggaa ggatctatac attgaatcaa tattggccat

181 tagccatatt attcattggt tatatatcat aaatcaatat tggctattgg ccattgcata

241 cgttgtatcc atatcataat atgtacattt atattggctc atgtccaaca ttaccgccat

301 gttgacattg attattgact agttattaat agtaatcaat tacggggtca ttagttcata

361 gcccatatat ggagttccgc gttacataac ttacggtaaa tggcccgcct ggctgaccgc

421 ccaacgaccc ccgcccattg acgtcaataa tgacgtatgt tcccatagta acgccaatag

481 ggactttcca ttgacgtcaa tgggtggagt atttacggta aactgcccac ttggcagtac

541 atcaagtgta tcatatgcca agtacgcccc ctattgacgt caatgacggt aaatggcccg

601 cctggcatta tgcccagtac atgaccttat gggactttcc tacttggcag tacatctacg

661 tattagtcat ccctattacc atggtgatgc ggttttggca gtacatcaat gggcgtggat

721 agcggtttca ctcacgggga tttccaagtc tccaccccat tgacgtcaat gggagtttgt

781 tttggcacca aaatcaacgg gactttccaa aatgtcgtaa caactccgcc ccattgacgc

841 aaatgggcgg taggcgtgta cggtgggagg tctatatagc agagctcgtt tagtgaaccg

901 tcagatctct agaagcttta atgcggtagt ttatcacagt taaattgcta acgcagtcag

961 gcaccgtgta tgaaatctaa caatgcgctc atcgtcatcc tcggcaccgt caccctggat

1021 gctctaggca taggcttggt tatgccggta ctgccgggcc tcttgcggga tatcgtccat

1081 tccgacagca tcgccagtca ctatggcgtg ctgctagcgc tatatgcgtt gatgcaattt

1141 ctatgcgcac ccgttctcgg agcactgtcc gaccgctttg gccgccgccc agtcctgctc

1201 gcttcgctac ttggagccac tatcgactac gcgatcatgg cgaccacacc cgtcctgtgg

1261 atccgtcgac ctgcagccaa gcttggcgta atcatggtca tagctgtttc ctgtgtgaaa

Transcription starts at position 901 (yellow). TATA-box (green) is at position 873. According to the manufacturer, a 363 nucleotide transcript is generated when using the ‘HeLa Nuclear Extract Positive Control DNA’ as a template for in vitro transcription.
