# Supplementary material for: A novel, non-radioactive eukaryotic in vitro transcription assay for sensitive quantification of RNA polymerase II activity
Source: BMC Mol Biol. 2014 Apr 3;15:7. doi: 10.1186/1471-2199-15-7 (PMC4021065; doi:10.1186/1471-2199-15-7)

Additional Figure S1 – Template comparison

Comparison of *in vitro* transcription assays using two different linear DNA templates, CMV-EGFP (E) or HS-DNA (H) in solution (free) or immobilized on magnetic beads (beads). Only positive transcription (+) and negative control reactions (-) were performed, and the product detected using primer extension and qPCR. E / H H_2O control = PCR control using the respective primer mix. **A.** After transcription, the beads were separated by centrifugation. The supernatant was collected and directly used for primer extension and qPCR. C(T) values and amplification curves did not allow to distinguish between the negative and positive reactions. **B**. The collected supernatant was used for RNA cleanup, and the reverse transcription and qPCR steps were performed using the purified RNA. All positive reactions showed positive amplification results, while for the negative transcription controls no amplification occurred. Best detection results were obtained when the bead-immobilized HS-DNA was used as a template.

A

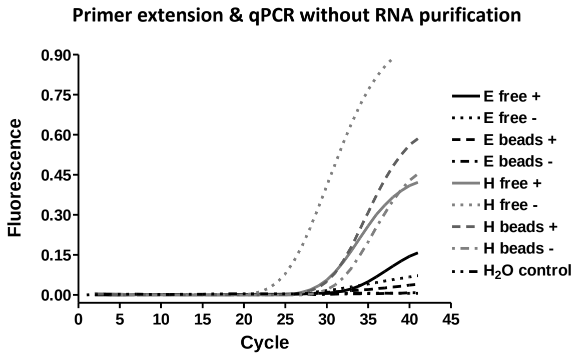

B

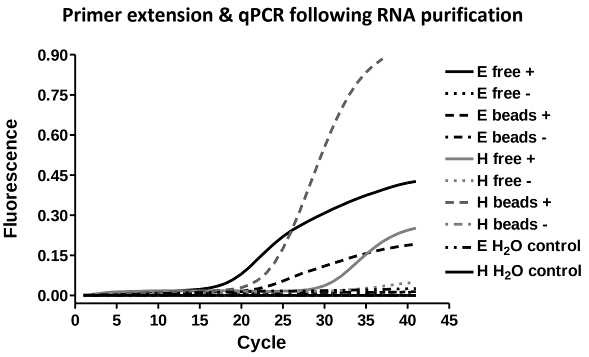

Supplement: Additional file 2 — Template comparison. qPCR amplification curves of the data presented in Table 2. [file 1471-2199-15-7-S2.pdf]
