# Supplementary material for: A novel, non-radioactive eukaryotic in vitro transcription assay for sensitive quantification of RNA polymerase II activity
Source: BMC Mol Biol. 2014 Apr 3;15:7. doi: 10.1186/1471-2199-15-7 (PMC4021065; doi:10.1186/1471-2199-15-7)
Supplement: Additional file 3 — Detailed Protocol. Detailed protocol for non-radioactive eukaryotic in vitro transcription and RNA quantification. [file 1471-2199-15-7-S3.pdf]

Detailed Protocol for Eukaryotic *In Vitro* Transcription and RNA Quantification

1. <u>Template DNA preparation and immobilization</u>

   a. Amplify the template DNA (**Btn-HN-DNA**) using the 'HeLa Nuclear Extract Positive Control DNA (CMV)' from the 'HeLaScribe® Nuclear Extract *in vitro* Transcription System' as a template.

      i. Dissolve and dilute the forward and reverse primers to 10µM each.
         Forward primer: Btn-HeLa_nuclear_fw
         5' Biotinyl –CTCATGTTTGACAGCTTATCGATCCGGGC 3'
         Reverse primer: HeLa_nuclear_rev 5' ACAGGACGGGTGTGGTCGCCATGAT 3'
         Dilute the template DNA to 200ng/µl

      ii. Set up the reaction mix:
          Per 50µl reaction: 25µl OneTaq Hotstart 2xMasterMix (standard buffer)
          2,5µl forward primer
          2,5µl reverse primer
          19µl H_2O
          Aliquot 49µl per reaction tube, add 1µl template DNA to positive reaction, 1µl H_2O to negative control.

      iii. Perform PCR:
           - Denaturation: 2min, 94°C
           - 30 cycles: denaturation 30s, 94°C
             annealing 30s, 60°C
             elongation 1min, 72°C
           - Final elongation: 10min, 72°C
           - Check amplification of correct length (1182bp) using gel electrophoresis.

   b. Purify the amplified DNA using a standard PCR purification kit. Do not use gel electrophoresis and gel-extraction! The dye used to visualize the DNA fragment would interfere with transcription. Check DNA concentration and dilute to 250µg/ml.

   c. Immobilize DNA onto Dynabeads M280

      i. Pre-treat the beads to remove RNase.
         - Transfer 500µl beads (10mg/ml) to a fresh tube, remove the supernatant using a magnet, resuspend the beads into 1000µl **Solution A** (0.1M NaOH, 0.05M KCl) and incubate the beads 2min in solution A. Repeat this step once.
         - Wash the beads once using 1000µl **Solution B** (0.1M KCl)
         - Wash the beads three times using 1000µl **1x B&W buffer** (5mM Tris-HCl pH 7.5; 0.5mM EDTA; 1M NaCl)

      ii. DNA binding:
          - Place the tube into a magnet and remove the supernatant. Resuspend the beads into 900µl **1x B&W buffer** and add 100µl of the **Btn-HN-DNA** (250µg/ml)
          - Incubate 15min at room temperature. Mix by gentle vortexing.
          - Place the tube into a magnet and transfer the supernatant to a fresh tube. Check DNA concentration and calculate the bead DNA loading yield. Typically a yield of 80% will be achieved.
          - Wash the DNA-beads three times using 1000µl **1x B&W buffer.**
          - Resuspend the DNA-beads into 1000µl **1x B&W buffer** and store at 4°C. This bead suspension at 5mg/ml beads will typically contain 20µg/ml DNA.

2. <u>Transcription reaction setup</u>

a. Planning for the reaction:
   i. Prepare additional transcription reaction buffer (20mM HEPES pH 7.9, 100mM KCl, 0.2mM EDTA, 0.5mM DTT, 20% glycerol) by diluting RNase-free solutions with water. Store the buffer in frozen aliquots.
   ii. Prepare a ribonucleotide mix containing 10mM ATP, 10mM CTP, 10mM GTP, 10mM UTP (NTP mix). Store in frozen aliquots.
   iii. Determine the volume of the 'HeLaScribe® Nuclear Extract' from the 'HeLaScribe® Nuclear Extract *in vitro* Transcription System' that corresponds to 8 activity units → **x**. Calculate the 'buffer volume' corresponding to **11µl-x**.

b. Before reaction setup:
   i. Calculate the DNA amount necessary to set up the desired number of reactions considering 150ng DNA per 25µl reaction. Resuspend the DNA-loaded beads and remove an aliquot that contains the desired amount of DNA. Typically, this will be 7.5µl DNA-bead suspension per reaction. Wash this aliquot two times in the double volume of transcription buffer using a magnet and resuspend for a third wash. Keep on ice until the inhibitor dilution series is prepared.
   ii. Prepare the transcription inhibitor dilutions as 5-fold of the desired final concentration. For example, prepare a 5µM amanitin solution in order to test α-amanitin at 1µM.

c. Reaction setup:
   i. Resuspend the DNA-beads in transcription buffer in a volume corresponding to the 'buffer volume', i.e. **11µl-x** per reaction.
   ii. Gently thaw a tube of extract on ice and mix by gentle pipetting. Transfer the required extract at **x µl** per reaction into the tube containing the beads.
   iii. Add following reaction components (volume per reaction) and mix by gentle vortexing:

RNase inhibitor (40U/µl)	1µl
$MgCl_2$ (50mM)	1.5µl
H_2O	2.5µl

   iv. Aliquot 19µl per reaction into the reaction tubes. Add 5µl of inhibitor dilution for the inhibited reactions or H_2O for the positive and negative reactions. Mix by gentle vortexing and incubate for 20min at room temperature.
   v. Add 1µl of the NTP mix to each tube except for the negative control. Mix by gentle vortexing and incubate for 30min at 30°C. During the incubation period, gentle vortex the reaction tubes 1-2 times to resuspend the beads.
   vi. Centrifuge the reaction tubes for 10min at 4°C and maximal speed. Continue with step 3A or 3B.

3A. RNA detection by primer extension and qPCR quantitation

a. RNA purification using RNeasy Mini kit
   i. Aliquot 400µl RLT buffer into fresh tubes.
   ii. After transcription and centrifugation, transfer 20µl of the bead supernatant to the RLT buffer. Mix by pipetting. Store the samples at -80°C until proceeding.
   iii. Thaw the samples. Add 250µl 100% EtOH and mix thoroughly.
   iv. Transfer the sample to an RNeasy column. Centrifuge or apply vacuum. Discard the flow-through.
   v. Apply 350µl buffer RW1 to the column. Centrifuge or apply vacuum. Discard the flow-through.
   vi. Prepare a DNase digestion mix using the Epicentre DNase Zero kit or alternative. Per column 160µl digestion mix containing 50 DNase units/ml will be needed.
   vii. Apply 160µl DNase digestion mix to the column. Incubate 15min at room temperature.
   viii. Apply 350µl buffer RW1 to the column. Centrifuge or apply vacuum. Discard the flow-through.
   ix. Apply 500µl buffer RPE supplemented with EtOH to the column. Centrifuge or apply vacuum. Discard the flow-through.
   x. Apply 500µl 80%EtOH to the column. Centrifuge or apply vacuum. Discard the flow-through.
   xi. Transfer the column to a dry collection tube. Centrifuge at maximal speed for 1min to completely dry the membrane.
   xii. Place the column into a fresh elution tube. Add 40µl H_2O to the column directly on the membrane. Incubate for 1min at room temperature. Elute by centrifugation.
   xiii. Repeat the elution step by adding another 40µl H_2O to the column, incubate and centrifuge.
   xiv. After freezing, place the RNA tubes into a speedvac until completely dry. Store the dried RNAs at -80°C.

b. DNase digestion
   i. Prepare 10µl DNase digestion mix per RNA sample using the Epicentre DNase Zero kit or alternative. Use 50U/ml DNase and 2U/µl RNase inhibitor.
   ii. Resuspend the dried RNA into 10µl digestion mix. Carefully wash the inside of the tube up to the 80µl level using the pipette to ensure that the complete RNA is dissolved.
   iii. Incubate for 30min at 37°C
   iv. Add 1.1µl stop reagent (or alternative).
   v. Incubate 10min at 65°C

c. Reverse transcription
   i. Prepare 7µl reverse transcription master mix per RNA sample using the Qiagen Sensiscript kit as follows:

      1µl Sensiscript buffer
      1µl Reverse primer HNrev1 10µM (5' CGGCCAAAGCGGTCGGACAGT 3')
      1µl dNTP mix, 500µM each
      0.5µl RNase inhibitor (40/µl)
      3.5µl H_2O

      Distribute 7µl aliquots of the mix into white qPCR tubes.
   ii. Add 3µl of the digested RNA sample.
   iii. Incubate for 60min at 37°C in a PCR machine, then cool down to 4°C. Keep at 4°C until ready for qPCR.

d. qPCR amplification

i. Prepare the 20x Probe-Primer-Mix H1 as follows:
20µl forward primer 100µM HNfrw1 (5' GCCGGGCCTCTTGCGGGATAT 3')
80µl reverse primer 100µM HNrev1 (5' CGGCCAAAGCGGTCGGACAGT 3')
10µl TaqMan® probe 100µM HN_SONDE1
(5' [6-FAM]TGGCGTGCTGCTAGCGCTAT[BHQ1] 3')
90µl H_2O

ii. Prepare a DNA standard curve (100pg/µl, 1pg/µl, 10fg/µl and 0.1fg/µl) using HN-DNA (amplified as Btn-HN-DNA but using the unbiotinylated forward primer). Transfer 10µl aliquots into white qPCR tubes. In an additional tube place 10µl H_2O for a PCR water control.

iii. Prepare 15µl qPCR master mix per RNA sample using the Sigma Jumpstart Readymix as follows:
12,5µl 2 x qPCR Mix
1,25µl $MgCl_2$ 50mM (final concentration 2,5mM)
1,25µl 20x Probe-Primer-Mix H1 (final concentrations: HNfrw1 500nM, HNrev1 2µM, HN_sonde1 250nM)
Transfer 15µl aliquots of the master mix to the reverse-transcribed samples, the standard curve and the water control. Mix and spin down.

iv. Perform qPCR
- Denaturation: 3min, 94°C
- 40 cycles: denaturation 15s, 94°C
  annealing 1min, 60°C
  elongation 1min, 72°C
  read plate
- Final: 4°C

v. Quantitate the RNA copy number in the samples using the standard curve. 1pg of HN-DNA contains 824,500 molecules.

3B. <u>RNA detection and quantification using the Quantigene assay</u>

a. RNA dilution
   i. After transcription and centrifugation, transfer 5µl of the bead supernatant to fresh tubes containing 95µl H_2O. Mix by vortexing. Store the samples at -80°C until proceeding.

b. Quantigene assay: Follow the instructions of the manufacturer!
   i. Pre-warm the Lysis Mixture to 37°C. Thaw the Blocking Reagent and centrifuge for 5min at 13.000 rpm. Thaw the customized oligonucleotide mix (Probe Set), mix and spin down. Pre-warm the hybridization oven at 55°C. Place the sealed capture plate for 30min at room temperature.
   ii. Prepare the working reagent for the desired number of wells as follows:

	1 well	50 wells
H_2O	60,4µl	3020µL
Lysis Mixture (37°C pre-warmed)	33,3µl	1665µL
Blocking Reagent (centrifuged!)	1µl	50µL
Probe Set	0,3µl	15µL
**Total:**	**95 µl**	**4750µL**

   iii. Distribute the working reagent mix as 95µl aliquots into the capture plate.
   iv. Add 5µl diluted reaction mix to each well containing working reagent. Avoid introducing air bubbles. If desired, an RNA standard curve can be also prepared to be used for signal quantification.
   v. Centrifuge the plate for 20s at 240 x g and room temperature.
   vi. Seal the plate and incubate overnight at 55°C.
   vii. Next day proceed with signal amplification and detection, as described in the Quantigene manual.
