# Supplementary material for: A novel, non-radioactive eukaryotic in vitro transcription assay for sensitive quantification of RNA polymerase II activity
Source: BMC Mol Biol. 2014 Apr 3;15:7. doi: 10.1186/1471-2199-15-7 (PMC4021065; doi:10.1186/1471-2199-15-7)
Supplement: Additional file 4 — Alignment of the HS-DNA, HS-DNA_mut and HS-DNA_long sequences. HS-DNA_mut is a mutant of the standard HS-DNA template containing a mutated TATA box. HS-DNA_long is a template similar to the standard HS-DNA template but including additional 98 bp within the run-off transcript (and primer extension/qPCR product) sequence. The sequence alignment highlights the differences between the three templates. [file 1471-2199-15-7-S4.pdf]

Sequence alignment of the PCR-derived DNA templates used in this study:

```
HS-DNA      CTCATGTTTGACAGCTTATCGATCCGGGCAACGTTGTTGCCATTGTCGCA    50
HS-DNA_mut  CTCATGTTTGACAGCTTATCGATCCGGGCAACGTTGTTGCCATTGTCGCA    50
HS-DNA_long CTCATGTTTGACAGCTTATCGATCCGGGCAACGTTGTTGCCATTGTCGCA    50

HS-DNA      GGCGGAGAACTGGTAGGTATGGAAGGATCTATACATTGAATCAATATTGG   100
HS-DNA_mut  GGCGGAGAACTGGTAGGTATGGAAGGATCTATACATTGAATCAATATTGG   100
HS-DNA_long GGCGGAGAACTGGTAGGTATGGAAGGATCTATACATTGAATCAATATTGG   100

HS-DNA      CCATTAGCCATATTATTCATTGGTTATATATCATAAATCAATATTGGCTA   150
HS-DNA_mut  CCATTAGCCATATTATTCATTGGTTATATATCATAAATCAATATTGGCTA   150
HS-DNA_long CCATTAGCCATATTATTCATTGGTTATATATCATAAATCAATATTGGCTA   150

HS-DNA      TTGGCCATTGCATACGTTGTATCCATATCATAATATGTACATTTATATTG   200
HS-DNA_mut  TTGGCCATTGCATACGTTGTATCCATATCATAATATGTACATTTATATTG   200
HS-DNA_long TTGGCCATTGCATACGTTGTATCCATATCATAATATGTACATTTATATTG   200

HS-DNA      GCTCATGTCCAACATTACCGCCATGTTGACATTGATTATTGACTAGTTAT   250
HS-DNA_mut  GCTCATGTCCAACATTACCGCCATGTTGACATTGATTATTGACTAGTTAT   250
HS-DNA_long GCTCATGTCCAACATTACCGCCATGTTGACATTGATTATTGACTAGTTAT   250

HS-DNA      TAATAGTAATCAATTACGGGGTCATTAGTTCATAGCCCATATATGGAGTT   300
HS-DNA_mut  TAATAGTAATCAATTACGGGGTCATTAGTTCATAGCCCATATATGGAGTT   300
HS-DNA_long TAATAGTAATCAATTACGGGGTCATTAGTTCATAGCCCATATATGGAGTT   300

HS-DNA      CCGCGTTACATAACTTACGGTAAATGGCCCGCCTGGCTGACCGCCCAACG   350
HS-DNA_mut  CCGCGTTACATAACTTACGGTAAATGGCCCGCCTGGCTGACCGCCCAACG   350
HS-DNA_long CCGCGTTACATAACTTACGGTAAATGGCCCGCCTGGCTGACCGCCCAACG   350

HS-DNA      ACCCCCGCCCATTGACGTCAATAATGACGTATGTTCCCATAGTAACGCCA   400
HS-DNA_mut  ACCCCCGCCCATTGACGTCAATAATGACGTATGTTCCCATAGTAACGCCA   400
HS-DNA_long ACCCCCGCCCATTGACGTCAATAATGACGTATGTTCCCATAGTAACGCCA   400

HS-DNA      ATAGGGACTTTCCATTGACGTCAATGGGTGGAGTATTTACGGTAAACTGC   450
HS-DNA_mut  ATAGGGACTTTCCATTGACGTCAATGGGTGGAGTATTTACGGTAAACTGC   450
HS-DNA_long ATAGGGACTTTCCATTGACGTCAATGGGTGGAGTATTTACGGTAAACTGC   450

HS-DNA      CCACTTGGCAGTACATCAAGTGTATCATATGCCAAGTACGCCCCCTATTG   500
HS-DNA_mut  CCACTTGGCAGTACATCAAGTGTATCATATGCCAAGTACGCCCCCTATTG   500
HS-DNA_long CCACTTGGCAGTACATCAAGTGTATCATATGCCAAGTACGCCCCCTATTG   500

HS-DNA      ACGTCAATGACGGTAAATGGCCCGCCTGGCATTATGCCCAGTACATGACC   550
HS-DNA_mut  ACGTCAATGACGGTAAATGGCCCGCCTGGCATTATGCCCAGTACATGACC   550
HS-DNA_long ACGTCAATGACGGTAAATGGCCCGCCTGGCATTATGCCCAGTACATGACC   550

HS-DNA      TTATGGGACTTTCCTACTTGGCAGTACATCTACGTATTAGTCATCCCTAT   600
HS-DNA_mut  TTATGGGACTTTCCTACTTGGCAGTACATCTACGTATTAGTCATCCCTAT   600
HS-DNA_long TTATGGGACTTTCCTACTTGGCAGTACATCTACGTATTAGTCATCCCTAT   600

HS-DNA      TACCATGGTGATGCGGTTTTGGCAGTACATCAATGGGCGTGGATAGCGGT   650
HS-DNA_mut  TACCATGGTGATGCGGTTTTGGCAGTACATCAATGGGCGTGGATAGCGGT   650
HS-DNA_long TACCATGGTGATGCGGTTTTGGCAGTACATCAATGGGCGTGGATAGCGGT   650

HS-DNA      TTCACTCACGGGGATTTCCAAGTCTCCACCCCATTGACGTCAATGGGAGT   700
HS-DNA_mut  TTCACTCACGGGGATTTCCAAGTCTCCACCCCATTGACGTCAATGGGAGT   700
HS-DNA_long TTCACTCACGGGGATTTCCAAGTCTCCACCCCATTGACGTCAATGGGAGT   700

HS-DNA      TTGTTTTGGCACCAAAATCAACGGGACTTTCCAAAATGTCGTAACAACTC   750
HS-DNA_mut  TTGTTTTGGCACCAAAATCAACGGGACTTTCCAAAATGTCGTAACAACTC   750
HS-DNA_long TTGTTTTGGCACCAAAATCAACGGGACTTTCCAAAATGTCGTAACAACTC   750
```

```
HS-DNA      CGCCCCATTGACGCAAATGGGCGGTAGGCGTGTACGGTGGGAGGTCTATA   800
HS-DNA_mut  CGCCCCATTGACGCAAATGGGCGGTAGGCGTGTACGGTGGGAGGTCTAGC   800
HS-DNA_long CGCCCCATTGACGCAAATGGGCGGTAGGCGTGTACGGTGGGAGGTCTATA   800

HS-DNA      TAGCAGAGCTCGTTTAGTGAACCGTCAGATCTCTAGAAGCTTTAATGCGG   850
HS-DNA_mut  TAGCAGAGCTCGTTTAGTGAACCGTCAGATCTCTAGAAGCTTTAATGCGG   850
HS-DNA_long TAGCAGAGCTCGTTTAGTGAACCGTCAGATCTCTAGAAGCTTTAATGCGG   850

HS-DNA      TAGTTTATCACAGTTAAATTGCTAACGCAGTCAGGCACCGTGTATGAAAT   900
HS-DNA_mut  TAGTTTATCACAGTTAAATTGCTAACGCAGTCAGGCACCGTGTATGAAAT   900
HS-DNA_long TAGTTTATCACAGTTAAATTGCTAACGCAGTCAGGCACCGTGTATGAAAT   900

HS-DNA      CTAACAATGCGCTCATCGTCATCCTCGGCACCGTCACCCTGGATGCTCTA   950
HS-DNA_mut  CTAACAATGCGCTCATCGTCATCCTCGGCACCGTCACCCTGGATGCTCTA   950
HS-DNA_long CTAACAATGCGCTCATCGTCATCCTCGGCACCGTCACCCTGGATGCTCTA   950

HS-DNA      GGCATAGGCTTGGTTATGCCGGTACTGCCGGG..................   982
HS-DNA_mut  GGCATAGGCTTGGTTATGCCGGTACTGCCGGG..................   982
HS-DNA_long GGCATAGGCTTGGTTATGCCGGTACTGCCGGGACGCAACTGAATGAAATG  1000

HS-DNA      ..................................................   982
HS-DNA_mut  ..................................................   982
HS-DNA_long GTGAAGGACGGGTCCAGGTGTGGCTGCTTCGGCAGTGCAGCTTGTTGAGT  1050

HS-DNA      ..............................CCTCTTGCGGGATATCGTCC  1002
HS-DNA_mut  ..............................CCTCTTGCGGGATATCGTCC  1002
HS-DNA_long AGAGTGTGAGCTCCGTAACTAGTCGCGTCGCCTCTTGCGGGATATCGTCC  1100

HS-DNA      ATTCCGACAGCATCGCCAGTCACTATGGCGTGCTGCTAGCGCTATATGCG  1052
HS-DNA_mut  ATTCCGACAGCATCGCCAGTCACTATGGCGTGCTGCTAGCGCTATATGCG  1052
HS-DNA_long ATTCCGACAGCATCGCCAGTCACTATGGCGTGCTGCTAGCGCTATATGCG  1150

HS-DNA      TTGATGCAATTTCTATGCGCACCCGTTCTCGGAGCACTGTCCGACCGCTT  1102
HS-DNA_mut  TTGATGCAATTTCTATGCGCACCCGTTCTCGGAGCACTGTCCGACCGCTT  1102
HS-DNA_long TTGATGCAATTTCTATGCGCACCCGTTCTCGGAGCACTGTCCGACCGCTT  1200

HS-DNA      TGGCCGCCGCCCAGTCCTGCTCGCTTCGCTACTTGGAGCCACTATCGACT  1152
HS-DNA_mut  TGGCCGCCGCCCAGTCCTGCTCGCTTCGCTACTTGGAGCCACTATCGACT  1152
HS-DNA_long TGGCCGCCGCCCAGTCCTGCTCGCTTCGCTACTTGGAGCCACTATCGACT  1250

HS-DNA      ACGCGATCATGGCGACCACACCCGTCCTGT  1182
HS-DNA_mut  ACGCGATCATGGCGACCACACCCGTCCTGT  1182
HS-DNA_long ACGCGATCATGGCGACCACACCCGTCCTGT  1280
```

Green: TATA-box
Blue: Mutated TATA box

Yellow: Start of transcription

Red: 98bp nucleotide insert in HS-DNA_long

Pink: Priming site for primer extension
